# The association between non-high-density lipoprotein cholesterol to high-density lipoprotein cholesterol ratio and chronic obstructive pulmonary disease: the mediating role of dietary inflammatory index

**DOI:** 10.3389/fnut.2024.1427586

**Published:** 2024-09-09

**Authors:** Ruying Wu, Hongyang Gong

**Affiliations:** ^1^Department of Surgery 3, Hebei Provincial First Veterans Hospital (Hebei General Hospital for Veterans), Xingtai, Hebei, China; ^2^Department of Physiology, College of Medicine, Chosun University, Gwangju, Republic of Korea

**Keywords:** NHHR, DII, COPD, NHANES, mediation analysis

## Abstract

**Background:**

Numerous studies have indicated a potential correlation between COPD, lipid metabolism, and dietary inflammation. However, the exact mechanisms by which dietary inflammation regulates the pathological processes of COPD related to lipid metabolism remain unclear. NHHR is a novel composite index of atherosclerotic lipid profiles, while the Dietary Inflammatory Index (DII) measures diet-induced inflammation. This study explores the relationship between NHHR and COPD and evaluates whether DII mediates this association.

**Methods:**

We employed multivariable logistic regression, smooth curve fitting, threshold effect analysis, and subgroup analysis to explore the relationship between NHHR and the incidence of COPD. Additionally, we conducted a mediation analysis to explore the potential relationship between dietary inflammatory index (DII) levels and the relationship between NHHR and COPD.

**Results:**

This analysis encompassed 13,452 participants, with 2,332 reporting incidents of COPD. Following adjustment for all covariates using multivariable logistic regression, each unit increase in NHHR level and DII level was associated with a 10% (OR = 1.10, 95% CI: 1.05, 1.16) and 8% (OR = 1.08, 95% CI: 1.04, 1.13) increase, respectively, in the incidence rate of COPD. Furthermore, compared to the lowest quartile, the highest quartile of NHHR level and DII level was associated with a 47% (*p* < 0.001) and 50% (*p* < 0.001) increase, respectively, in the incidence rate of COPD. Smooth curve fitting and threshold effect analysis revealed a nonlinear relationship between NHHR and the risk of COPD, with a breakpoint at 2.60. Mediation analysis indicated that DII mediated 7.24% of the association between NHHR and COPD (*p* = 0.004).

**Conclusion:**

Higher NHHR levels are associated with an increased prevalence of COPD. Moreover, this association is mediated by DII, suggesting that an anti-inflammatory diet may be beneficial.

## Introduction

COPD is a common chronic airway disorder characterized by persistent airflow limitation and associated respiratory symptoms. Clinical manifestations include chronic cough, sputum production, and progressively worsening dyspnea (1, 2). COPD has become an increasingly significant cause of morbidity, disability, and mortality globally, posing a severe threat to human health. Reports indicate that over 3 million people die from COPD annually worldwide (3), imposing a substantial burden on global public health. Therefore, the identification and management of modifiable risk factors are paramount in preventing or delaying the onset of COPD and reducing its associated complications.

Observational studies suggest that patients with hyperlipidemia are 1.48 times more likely to develop COPD compared to participants without hyperlipidemia (HR = 1.48, 95% CI: 1.44–1.53, *p* < 0.001) (4), yet the specific mechanisms remain uncertain. Furthermore, research indicates that alterations in lipid metabolism can lead to the production of inflammatory mediators, thereby contributing to the onset and progression of COPD (5). The ratio of non-high-density lipoprotein cholesterol (non-HDL-C) to high-density lipoprotein cholesterol (HDL-C) (NHHR) is a novel composite index for assessing the lipid profile that causes atherosclerosis (6), providing comprehensive insights into anti-atherogenic and atherogenic lipid particles (7). Previous studies have shown that NHHR is associated with many chronic diseases, such as diabetes (8), fatty liver (9), and kidney disease (10). Researcher Zhang et al. (11) and colleagues showed, based on a nationwide study of a large sample of 8,180 individuals aged 20–64 years, that DII levels were significantly and positively associated with Triglycerides, Waist circumference, and BMI levels, with beta and 95% CIs of 2.795 (1.003, 4.588), 0.881 (0.677, 1.084), and 0.318 (0.232, 0.405), respectively. And DII level was significantly negatively correlated with HDL-C level (beta = −0.803, 95%CI: −0.986, −0.621). Another cross-sectional study in adolescents aged 12–18 years (12) showed that DII levels were significantly and positively correlated with LDL-C levels (beta = 4.84, 95%CI: 0.05, 9.62), whereas they were positively but non-significantly correlated with Cholesterol, Triglycerides, and Waist circumference. In addition, dietary factors play an important role in modulating chronic inflammation (13, 14).

In summary, we hypothesize that the relationship between NHHR levels and the odds of developing COPD may be mediated by dietary inflammation. Moreover, pro-inflammatory dietary habits (DII score ≥ 0) have been associated with an increased risk of COPD and declining lung function (15, 16). Findings from a nationally representative study in the United States (17) demonstrate a significant positive correlation between higher DII scores and increased risk of COPD. Consequently, we speculate that dietary inflammation might contribute to the link between NHHR and COPD.

## Methods

### Study participants

The National Health and Nutrition Examination Survey (NHANES) is a continuous, stratified, multistage sampling program designed to assess the health and nutritional status of both adults and children in the United States, covering a wide range of health and nutrition measures. The NHANES research project has obtained approval from the Research Ethics Review Board of the National Center for Health Statistics (NCHS), and all participants have provided written informed consent (18).

Among the 70,190 participants across seven NHANES cycles from 2005 to 2018, there were 39,749 participants aged 20 years and older. After excluding participants with missing NHHR indicator data, missing DII data (*n* = 10,299), and missing COPD indicator data (*n* = 15,998) from the total of 39,749 participants, a final cohort of 13,452 participants was included in the study (Supplementary Figure S1).

### Definition of NHHR and DII

The independent variable in this study is NHHR, which, according to previous research (19), is defined as the ratio of Non- High-density lipoprotein cholesterol to High-density lipoprotein cholesterol. Non- High-density lipoprotein cholesterol is calculated as the total cholesterol minus High-density lipoprotein cholesterol.

The Dietary Inflammatory Index (DII) is a key indicator used to evaluate the inflammatory potential of a wide range of dietary components, spanning from vitamins to minerals (20). A DII score of ≥0 signifies a pro-inflammatory diet, whereas a DII score of <0 indicates an anti-inflammatory diet. Additionally, higher DII scores signify unhealthy dietary patterns, whereas lower DII scores denote healthier dietary patterns. The specific algorithm for calculating the DII score is provided in Supplementary material.

### Definition of COPD

According to previous studies (21, 22), COPD is defined as meeting one of the following criteria: (1) Post-bronchodilator FEV1/FVC < 0.7; (2) Self-reported emphysema or COPD; (3) Participants aged over 40 years with chronic bronchitis or a smoking history, who were also prescribed COPD medications such as mast cell stabilizers, leukotriene modifiers, inhaled corticosteroids, and selective phosphodiesterase-4 inhibitors.

### Covariables

According to previous studies (21, 22), the study covariates include age, gender, ethnicity, Married or not, education level, poverty status, obesity, smoking status, alcohol consumption, and sleep disorders. Additionally, the Charlson Comorbidity Index (CCI) was employed to evaluate participants’ health status and mitigate multicollinearity among comorbidities (23). For detailed information regarding these covariates, please refer to Supplementary Tables S1, S2.

### Statistical analysis

Sampling weights were utilized in all statistical analyses to ensure the estimated data’s national representativeness. In our study, “WTMEC2YR” was employed as the weighting variable, with the new weights (2005–2018) calculated as 1/7 × WTMEC2YR. Continuous variables are expressed as mean ± standard deviation (SD), and categorical variables are presented as frequencies (percentages) (24). Weighted t-tests were performed for continuous variables, and weighted chi-square tests were conducted for categorical variables to evaluate differences between the Non-COPD and COPD groups. Weighted multivariable logistic regression was used to evaluate the relationship between NHHR, DII, and COPD. Weighted linear regression was utilized to assess the relationship between NHHR and DII. To control for confounding factors, we adjusted for the following factors in the regression models (Model 1 was unadjusted for any potential confounders. Model 2 was adjusted for age, gender, race, Married or not, education level, and poverty status; Model 3 further adjusted for sleep disorders, obesity, smoking status, alcohol consumption status, and CCI). Results are presented as odds ratios (ORs) or β coefficients with their respective 95% confidence intervals (95% CIs). Additionally, subgroup analyses were conducted to explore potential relationships between NHHR and COPD across various subgroups. Restricted cubic splines (RCS) and segmented linear regression models were used to investigate potential nonlinear association and threshold effects between NHHR and COPD (25). To examine whether the effect of NHHR (X) on COPD (Y) occurrence was mediated by DII (M), mediation analysis was performed based on the precondition of “statistically significant association between X and M” and “statistically significant association between M and Y” (26).

Several sensitivity analyses were performed in this study to verify the robustness of the results. First, Multiple imputation by chained equations (MICE) and repeated the main analyses (27). We used multiple imputations, based on 5 imputed data sets to calculate missing NHHR, DII, and COPD data. In addition, we excluded subjects with asthma for further analyses. Finally, to further control for confounders, we further adjusted for Triglycerides, LDL-C, HDL-C, LDL-C, and glycohemoglobin. We used the vif() function from the “car “package to test for multicollinearity in the covariates, with a variance inflation factor (VIF) < 10 indicating the absence of multicollinearity (28). In the present study, all the VIFs were less than 2. The mediation effect was determined using the “mediation” package in R software (29). All data analyses were conducted in R software (version 4.3.1), utilizing the “survey” and “ggplot2” packages for weighted data analysis, restricted cubic spline functions, and visualization. A two-sided *p*-value <0.05 was considered statistically significant (30).

## Results

### Baseline characteristics

In this study, a total of 13,452 participants aged 20 years or older were included, representing approximately 80.01 million US adults. The mean (SD) NHHR value was 2.85 (1.46), and the prevalence of COPD was 17.34% (equivalent to 13.85 million individuals). Additionally, both NHHR and DII levels were higher in the COPD group compared to the Non-COPD group. Preliminary assessment revealed that individuals in the COPD group were characterized by older age, female gender, White race, married status, higher socioeconomic status, obesity, smoking, alcohol consumption, higher CCI, and higher DII scores compared to those in the non-COPD group. For specific details, please refer to Table 1.

**Table 1:** Baseline characteristics of participants with or without COPD.

Characteristic	Total	Non-COPD	COPD	*p*-value
Prevalence, weighted N, in millions (%)	86.01	72.16	13.85	
No. of participants in the sample	13,452	11,120	2,332	
Age, weighted N, in millions (%)				
20–40	29.65 (34.47)	27.11 (37.58)	2.54 (18.29)	<0.001
41–60	31.88 (37.07)	26.53 (36.77)	5.35 (38.64)	
>60	24.48 (28.46)	18.51 (25.66)	5.97 (43.06)	
Gender, weighted N, in millions (%)				
Male	40.29 (46.84)	34.86 (48.31)	5.43 (39.17)	<0.001
Female	45.72 (53.16)	37.30 (51.69)	8.42 (60.83)	
Race, weighted N, in millions (%)				
Mexican American	6.67 (7.75)	6.25 (8.66)	0.42 (3.04)	<0.001
Non-Hispanic White	58.49 (68.01)	47.64 (66.02)	10.85 (78.37)	
Non-Hispanic Black	8.98 (10.44)	7.78 (10.78)	1.20 (8.72)	
Other	11.87 (13.80)	10.50 (14.55)	1.37 (9.88)	
Marital status, weighted N, in millions (%)				
No	30.93 (35.97)	25.16 (34.87)	5.77 (41.68)	<0.001
Yes	55.07 (64.03)	46.99 (65.13)	8.08 (58.32)	
Education, weighted N, in millions (%)				
Below high school	11.09 (12.89)	8.33 (11.55)	2.76 (19.89)	<0.001
High School or above	74.92 (87.11)	63.82 (88.45)	11.10 (80.11)	
PIR, weighted N, in millions (%)				
Not poor	63.23 (79.23)	54.19 (81.05)	9.04 (69.80)	<0.001
poor	16.58 (20.77)	12.67 (18.95)	3.91 (30.20)	
Obesity, weighted N, in millions (%)				
No	50.02 (58.53)	42.91 (59.78)	7.11 (51.98)	<0.001
Yes	35.44 (41.47)	28.87 (40.22)	6.57 (48.02)	
Smoking, weighted N, in millions (%)				
Never	47.41 (55.12)	43.28 (59.98)	4.13 (29.81)	<0.001
Former	22.47 (26.12)	17.69 (24.51)	4.78 (34.52)	
Current	16.13 (18.76)	11.19 (15.51)	4.94 (35.67)	
Drinking, weighted N, in millions (%)				
No	17.79 (21.79)	14.46 (21.18)	3.33 (24.90)	0.012
Yes	63.84 (78.21)	53.79 (78.82)	10.05 (75.10)	
Sleep disorder, weighted N, in millions (%)				
No	65.12 (75.76)	55.75 (77.33)	9.37 (67.61)	<0.001
Yes	20.83 (24.24)	16.35 (22.67)	4.48 (32.39)	
CCI [mean (SD)]				
<1	44.56 (51.81)	41.05 (56.90)	3.51 (25.32)	<0.001
≥1	41.45 (48.19)	31.10 (43.10)	10.35 (74.68)	
NHHR [mean (SD)]	2.85 (1.46)	2.80 (1.43)	3.07 (1.63)	<0.001
DII [mean (SD)]	1.04 (1.71)	0.95 (1.71)	1.47 (1.62)	<0.001

Mean (SD) for continuous variables: the P value was calculated by the weighted linear regression model. Percentages (weighted N, in millions) for categorical variables: the P value was calculated by the weighted chi-square test. NHHR, Non-high-density lipoprotein cholesterol (non-HDL-C) to high-density lipoprotein cholesterol (HDL-C) ratio; COPD, Chronic obstructive pulmonary disease; DII, dietary inflammatory index; CCI, Charlson Comorbidity Index; PIR, Ratio of family income to poverty.

### The association between NHHR and COPD

As shown in Table 2, three different models were employed to assess the association between NHHR and COPD. In model 3 adjusting for all covariates, for every 1 standard deviation increase in NHHR, there was a 10% increase in the odds of COPD [1.10 (1.05, 1.16)]. Furthermore, compared to the first quartile (Q1) of NHHR, the odds of COPD increased by 47% in the fourth quartile (Q4) [1.47 (1.19, 1.81)] in the model 3. Additionally, as NHHR increases from Q1 to Q4, the OR values also gradually increase, with a P for trend less than 0.001. Models 1 and 2 also demonstrate consistency in the results.

**Table 2:** Association between NHHR, DII, and COPD.

Characteristics	Model 1 [OR (95% CI)]	*P*	Model 2 [OR (95% CI)]	*P*	Model 3 [OR (95% CI)]	*P*
NHHR						
Continues	1.12 (1.07, 1.16)	<0.001	1.17 (1.12, 1.23)	<0.001	1.10 (1.05, 1.16)	<0.001
Q1	1 (ref.)		1 (ref.)		1 (ref.)	
Q2	1.19 (0.99, 1.44)	0.064	1.22 (0.99, 1.51)	0.061	1.10 (0.88, 1.37)	<0.001
Q3	1.20 (0.97, 1.48)	0.085	1.28 (1.02, 1.61)	0.035	1.15 (0.91, 1.46)	0.272
Q4	1.63 (1.37, 1.94)	<0.001	1.92 (1.59, 2.33)	<0.001	1.47 (1.19, 1.81)	<0.001
P for trend	<0.001		<0.001		<0.001	
DII						
Continues	1.21 (1.16, 1.26)	<0.001	1.15 (1.10, 1.20)	<0.001	1.08 (1.04, 1.13)	<0.001
Q1	1 (ref.)		1 (ref.)		1 (ref.)	
Q2	1.52 (1.23, 1.87)	<0.001	1.49 (1.20, 1.86)	<0.001	1.30 (1.10, 1.75)	0.007
Q3	1.53 (1.27, 1.86)	<0.001	1.35 (1.12, 1.63)	0.002	1.17 (0.95, 1.43)	0.140
Q4	2.36 (1.90, 2.93)	<0.001	1.95 (1.55, 2.44)	<0.001	1.50 (1.19, 1.89)	<0.001
P for trend	<0.001		<0.001		0.003	

Model 1: no covariates were adjusted. Model 2: age, gender, education level, marital, PIR, and race were adjusted. Model 3: age, gender, education level, marital, PIR, race, obesity, smoking, drinking, sleep disorder, and CCI were adjusted. NHHR, Non-high-density lipoprotein cholesterol (non-HDL-C) to high-density lipoprotein cholesterol (HDL-C) ratio; COPD, Chronic obstructive pulmonary disease; DII, dietary inflammatory index; CCI, Charlson Comorbidity Index; PIR, Ratio of family income to poverty; OR, odds ratio; CI, confidence interval.

**Figure 1:**
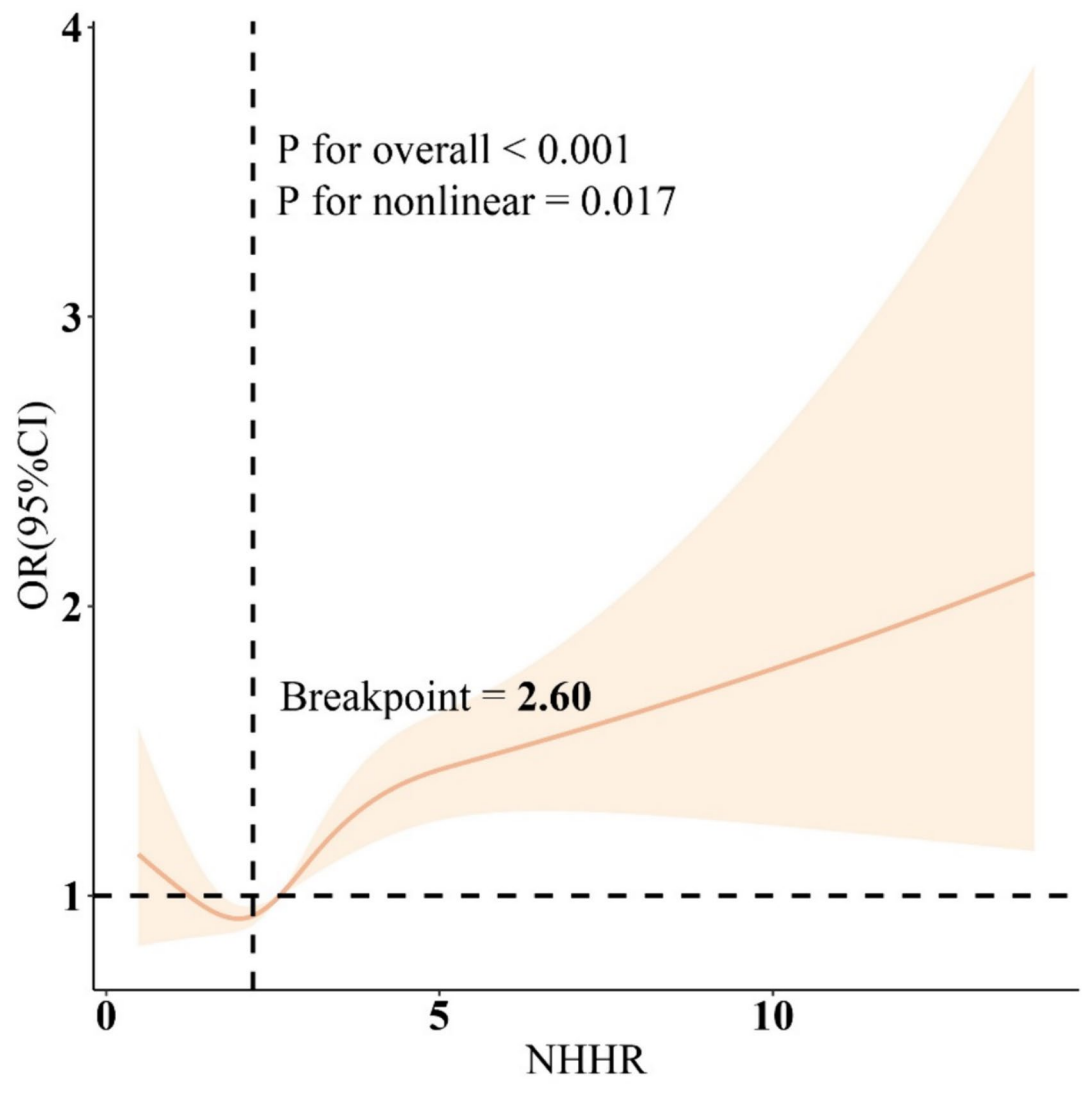
Restricted cubic spline curves for the association between the NHHR and COPD. Adjusted for age, gender, education level, marital, PIR, race, obesity, smoking, drinking, sleep disorder, and CCI. NHHR, Non-high-density lipoprotein cholesterol (non-HDL-C) to high-density lipoprotein cholesterol (HDL-C) ratio; COPD, Chronic obstructive pulmonary disease; CCI, Charlson Comorbidity Index; PIR, Ratio of family income to poverty.

**Figure 2:**
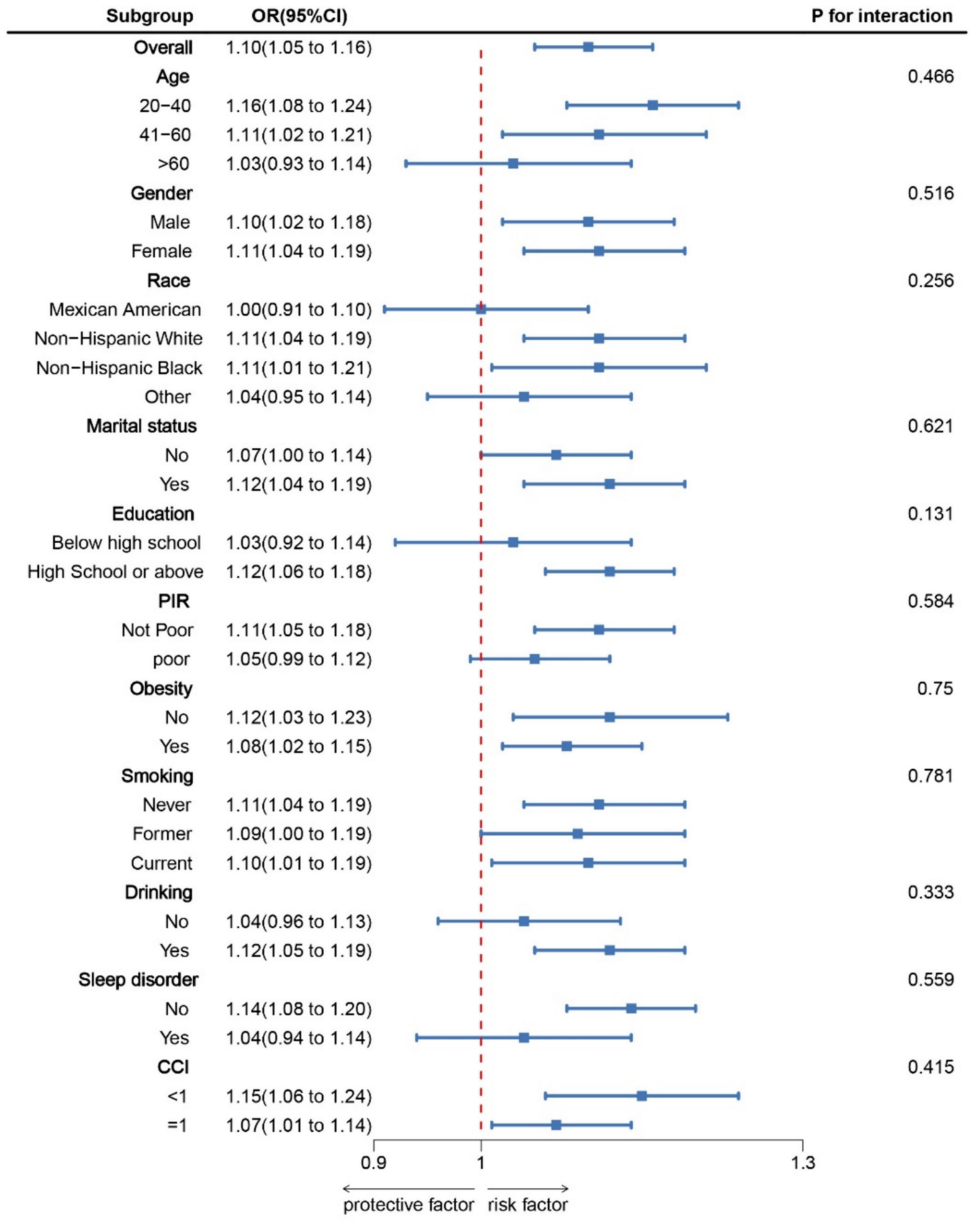
Subgroup analysis for the association between NHHR and COPD. OR was calculated as each standard deviation increase in NHHR. Adjusted for age, gender, education level, marital, PIR, race, obesity, smoking, drinking, sleep disorder, and CCI. NHHR, Non-high-density lipoprotein cholesterol (non-HDL-C) to high-density lipoprotein cholesterol (HDL-C) ratio; COPD, Chronic obstructive pulmonary disease; CCI, Charlson Comorbidity Index; PIR, Ratio of family income to poverty.

The RCS results (Figure 1) revealed a J-shaped nonlinear association between NHHR and COPD, with an inflection point at 2.60 (P for non-linear = 0.017). Threshold effect analysis indicated that when NHHR levels were below 2.60, there was no significant association between NHHR and COPD [OR = 1.05, 95% CI: 0.81, 1.36]. However, when NHHR levels exceeded 2.60, there was a significant positive relationship between NHHR and COPD [OR = 1.09(1.02, 1.16)] (Table 3). Subgroup analysis demonstrated that the correlation between NHHR and COPD risk remained robust across all subgroups (Figure 2).

**Table 3:** Threshold effect analysis of NHHR on COPD using the two-piecewise linear regression model.

Adjusted	OR (95%CI), *P*-value
Fitting model by standard linear regression	1.10 (1.05, 1.16), <0.001
Fitting model by two-piecewise linear regression	
Inflection point	2.60
NHHR<2.60	1.05 (0.81, 1.36), 0.700
NHHR>2.60	1.09 (1.02, 1.16), 0.009
P for the likelihood ratio test	<0.001

Adjusted for age, gender, education level, marital, PIR, race, obesity, smoking, drinking, sleep disorder, and CCI.

### DII and risk of COPD

Table 2 displays the association between DII and COPD. In model 3, Following adjustment for all covariates, compared to the first quartile (Q1) of DII, the odds of COPD increased by 50% in the fourth quartile (Q4) [OR = 1.50 (1.19, 1.89)]. When DII was considered as a continuous variable, the positive relationship between DII and COPD remained statistically significant (OR = 1.08, 95% CI: 1.04, 1.13). Models 1 and 2 also demonstrate consistency in the results. Additionally, the RCS results (Supplementary Figure S2) revealed a significant linear association between DII and COPD.

### Association of NHHR and DII

Following adjustment for all covariates, there was a significant statistical association between NHHR and DII (β = 0.10, 95% CI: 0.07, 0.12, *p* < 0.001) (Supplementary Table S3).

### Mediation effect

**Figure 3:**
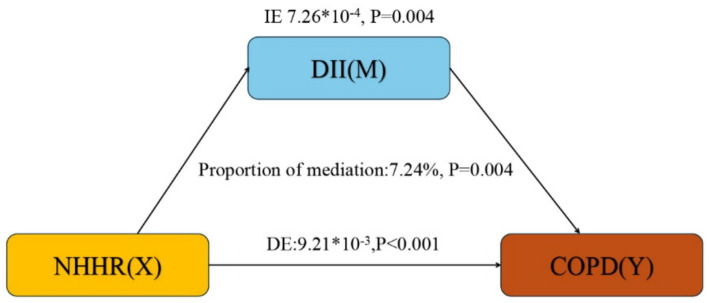
Mediation analysis. IE, indirect effect; DE, direct effect; NHHR, Non-high-density lipoprotein cholesterol (non-HDL-C) to high-density lipoprotein cholesterol (HDL-C) ratio; COPD, Chronic obstructive pulmonary disease; DII, dietary inflammatory index.

Based on the above analysis, our study meets the prerequisites for conducting mediation analysis. Following adjustment for all covariates, we observed the mediation effect of DII (Figure 3). DII (indirect effect = 7.26*10^−4^, *p* = 0.004; direct effect = 9.21*10^−3^, *p* < 0.001) mediated 7.24% (mediation proportion = indirect effect/(indirect effect + direct effect) *100%, *p* = 0.004) of the association between NHHR and COPD risk. Therefore, DII can be considered a mediating factor in the relationship between NHHR and the odds of COPD.

### Sensitivity analysis

To ensure the stability of the results, this study performed multiple interpolations of missing data for NHHR, DII, and COPD, which remained consistent with the primary results (Supplementary Tables S4, S5). In addition, the results remained robust after excluding participants with asthma (Supplementary Tables S6, S7). After further adjustments for Triglycerides, LDL-C, HDL-C, LDL-C, and Glycohemoglobin, the results remained stable (Supplementary Table S8).

## Discussion

This study provides new insights into the mediating role of DII in the association between NHHR and COPD. Our results indicate that participants with elevated NHHR and DII are at increased risk of COPD. Additionally, it is noteworthy that we identified a J-shaped association between NHHR and COPD, with a turning point at 2.60. These findings suggest that NHHR could potentially function as a predictive factor for COPD development, and anti-inflammatory diets may mitigate the impact of NHHR on COPD risk.

In recent years, there has been increasing scholarly attention to the role of inflammatory dietary patterns in the progression of COPD. In a cross-sectional study based on a national survey involving 9,929 American participants, 5.69% of individuals were diagnosed with COPD, and high DII levels were identified as an independent risk factor for COPD (17). Similarly, in our study, we observed similar results, indicating a positive correlation between DII and the occurrence of COPD. Furthermore, findings from a study involving 4,267 Korean men aged over 40 also demonstrated that DII was significantly associated with lung function and COPD (31). Promoting a healthy diet with anti-inflammatory properties may help prevent chronic obstructive pulmonary disease in adult men. Our study once again highlights the potential role of DII in COPD.

An increasing body of research indicates that NHHR can predict the risk of most lipid-related diseases (32, 33). Although direct studies on the connection between COPD and lipid metabolism are relatively scarce at present, most studies have shown a potential relationship between COPD and various lipid-related factors. For instance, a prospective cohort study with a 5-year follow-up found that for every 100 mg increase in triglyceride levels, the mortality rate of COPD patients increased by 42% (HR: 1.42, 95% CI: 1.06–1.89) (34). Additionally, a retrospective cohort study conducted in Taiwan, spanning over a decade, included 21,790 patients with hyperlipidemia and 87,160 patients without hyperlipidemia. It found that the risk of COPD occurrence was 1.48 times higher among patients with hyperlipidemia compared to those without (95% CI, 1.44, 1.53) (4). These findings indirectly support a positive correlation between NHHR levels and COPD occurrence, further aiding researchers in exploring the potential associations between lipid profiles, such as NHHR, and COPD incidence.

This study indicates that low levels of DII scores (representing a healthy diet) may mitigate the relationship between NHHR and the likelihood of COPD. Research by Fekete et al. (35) suggests that COPD patients often have inadequate intake of omega-3 polyunsaturated fatty acids (PUFAs), which have anti-inflammatory properties and can lower cholesterol and triglyceride levels. Our study corroborates this viewpoint. On the other hand, higher DII levels are associated with elevated total cholesterol (TC) and LDL-C levels (36), and lipid metabolism disorders exacerbate the occurrence and progression of COPD (37). Thus, our study findings are further supported: an anti-inflammatory dietary pattern (i.e., lower DII scores) helps alleviate the relationship between NHHR and the likelihood of COPD.

This study found a potential link between DII and the relationship between NHHR and COPD. Firstly, the interaction between inflammation and lipid metabolism is notable. COPD is characterized by airflow limitation and chronic airway inflammation. Inflammation is prevalent in COPD patients and is closely related to lipid metabolism disorders (38). Inflammatory mediators, such as tumor necrosis factor-α (TNF-α), interleukin-6 (IL-6), and C-reactive protein (CRP), are not only elevated in the local airways but also in the systemic circulation (39). Moreover, these mediators can affect lipid metabolism through various pathways: (1) Inflammatory mediators promote adipocyte catabolism: TNF-α and IL-6 can induce adipocytes to secrete fatty acids and reduce fat synthesis. This metabolic change leads to increased plasma free fatty acids (FFA), further causing insulin resistance and lipid metabolism disorders (40). (2) Inflammatory mediators influence cholesterol metabolism: During inflammation, the liver synthesizes acute-phase response proteins such as CRP and serum amyloid A (SAA), which bind to high-density lipoprotein (HDL), impairing HDL function and affecting cholesterol transport and reverse cholesterol transport (41). Secondly, lipid metabolism disorders impact lung function. These disorders affect overall health and can directly impact lung function. Specifically: (1) Lipid deposition and airway structural changes: Abnormal lipid deposition in airway smooth muscle and surrounding tissues may lead to airway wall thickening and airflow limitation, exacerbating COPD’s pathological changes (42). (2) Oxidative stress and cellular damage: Elevated plasma FFA and low-density lipoprotein (LDL) can be oxidized to form oxidized LDL (ox-LDL), which induces oxidative stress and apoptosis in lung tissue, further damaging the lungs (43). Lastly, diet regulates inflammation and lipid metabolism. Dietary patterns play a critical role in modulating these processes, and appropriate dietary interventions can alleviate inflammation and lipid metabolism disorders in COPD patients. For example: (1) Anti-inflammatory diet: Foods rich in omega-3 fatty acids (such as fish oil) have anti-inflammatory effects and can reduce the production of inflammatory mediators (44, 45). Additionally, foods rich in polyphenols (such as fruits and vegetables) have significant anti-inflammatory effects (46). (2) Lipid-regulating diet: A low-fat diet rich in fiber and antioxidants can improve lipid metabolism, lower plasma FFA and LDL levels, and help reduce the risk and progression of COPD (47, 48).

Our research possesses the following advantages: (1) NHHR demonstrates its unique predictive accuracy across multiple studies. Additionally, NHHR, characterized by its non-invasiveness, novelty, simplicity, accessibility, and cost-effectiveness, is widely adopted as a clinical predictive factor, bringing extensive developmental prospects to the clinical practice of COPD patients (19). (2) We utilize nationally representative data and consider sample weights, facilitating a comprehensive exploration of the relationship between NHHR in U.S. adults and the likelihood of COPD. (3) To the best of our knowledge, this is the first investigation of the mediating role of DII scores in the association between NHHR and the odds of COPD.

However, our study also has some limitations: (1) While the mediation analysis reflects NHHR’s potential influence on COPD occurrence through DII, extensive prospective cohort studies, randomized controlled trials, or animal experiments are still needed in the future to establish conclusive pathogenic mechanisms. (2) Despite including a relatively large number of covariates based on previous research to enhance the robustness of our study results, limitations of the NHANES database prevent us from eliminating all potential confounding factors’ ultimate impact on the study results. Therefore, the results of this study should be interpreted cautiously and objectively. (3) The use of 24-h dietary recall and COPD-related questionnaires may introduce bias into the results, as they may not accurately reflect participants’ long-term or typical dietary indicators and COPD diagnosis.

## Conclusion

In conclusion, higher NHHR levels increase the risk of COPD, while lower DII levels may mitigate this relationship. Therefore, controlling NHHR levels and adhering to anti-inflammatory diets are of significant clinical value in reducing the risk of COPD.

## Data Availability

The datasets presented in this study can be found in online repositories. The names of the repository/repositories and accession number(s) can be found in the article/Supplementary material.
