# Supplementary material for: The association between non-high-density lipoprotein cholesterol to high-density lipoprotein cholesterol ratio and chronic obstructive pulmonary disease: the mediating role of dietary inflammatory index
Source: Front Nutr. 2024 Sep 9;11:1427586. doi: 10.3389/fnut.2024.1427586 (PMC11416962; doi:10.3389/fnut.2024.1427586)
Supplement: Supplementary file 1 [file Data_Sheet_1.docx]

**Supplementary Material**

**Calculation of the dietary inflammatory index**[1].

Calculation of the DII is based on dietary intake data that are then linked to the regionally representative world database that provided a robust estimate of a mean and standard deviation for each parameter. These then become the multipliers to express an individual's exposure relative to the ‘standard global mean’ as a Z-score. This is achieved by subtracting the ‘standard mean’ from the amount reported and dividing this value by its standard deviation. To minimize the effect of ‘right skewing’, this value is converted to a percentile score. To achieve a symmetrical distribution with values centred on 0 (null) and bounded between −1 (maximally anti-inflammatory) and +1 (maximally pro-inflammatory), each percentile score is doubled and then ‘1’ is subtracted.

The centred percentile value for each food parameter is then multiplied by its respective ‘overall food parameter-specific inflammatory effect score’ to obtain the ‘food parameter-specific DII score’. Finally, all of the ‘food parameter-specific DII scores’ are summed to create the ‘overall DII score’ for an individual.

The 28 nutrients in this study were alcohol, β-carotene, caffeine, carbohydrate, cholesterol, energy, total fat, fiber, folic acid, iron, magnesium, monounsaturated fatty acids, polyunsaturated fatty acids, n-3 fatty acids, n-6 fatty acids, protein, saturated fat, selenium, zinc, vitamin A, B1, B2, B3, B6, B12, C, D, and E[2].

1. Shivappa N, Steck SE, Hurley TG, Hussey JR, Hébert JR. Designing and developing a literature-derived, population-based dietary inflammatory index. Public Health Nutrition. 2014;17:1689–96.

2. Meng X, Sha W, Lou X, Chen J. The relationship between dietary inflammatory index and osteoporosis among chronic kidney disease population. Sci Rep. 2023;13:22867.

**
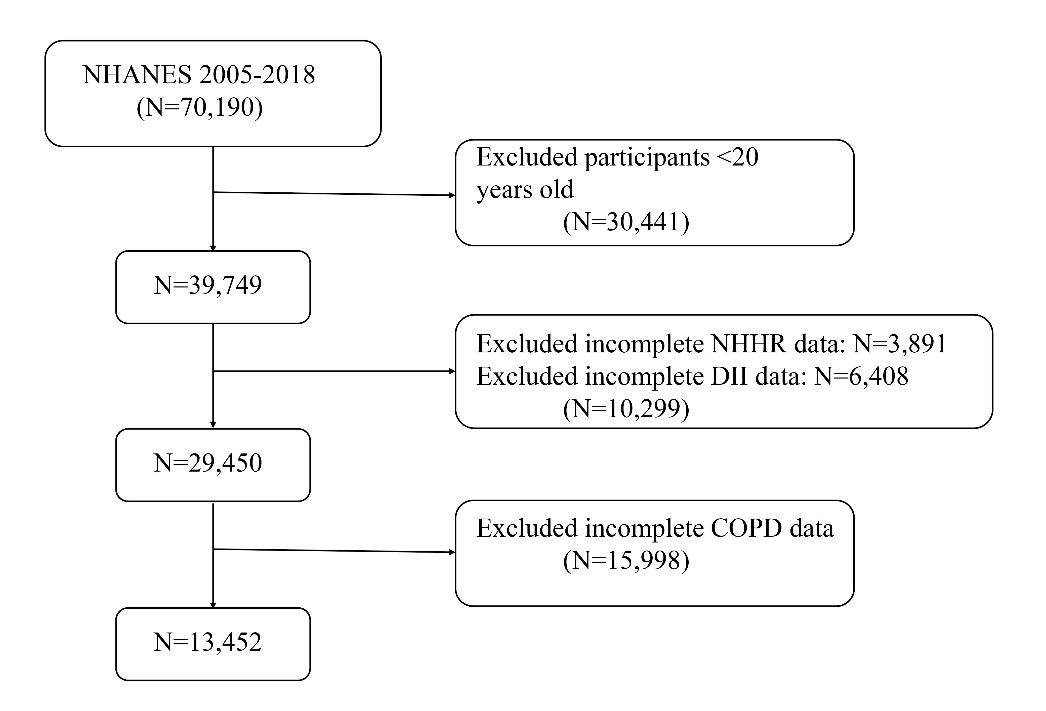
**

**Figure S1.** A flow diagram of eligible participant selection in the National Health and Nutrition Examination Survey.


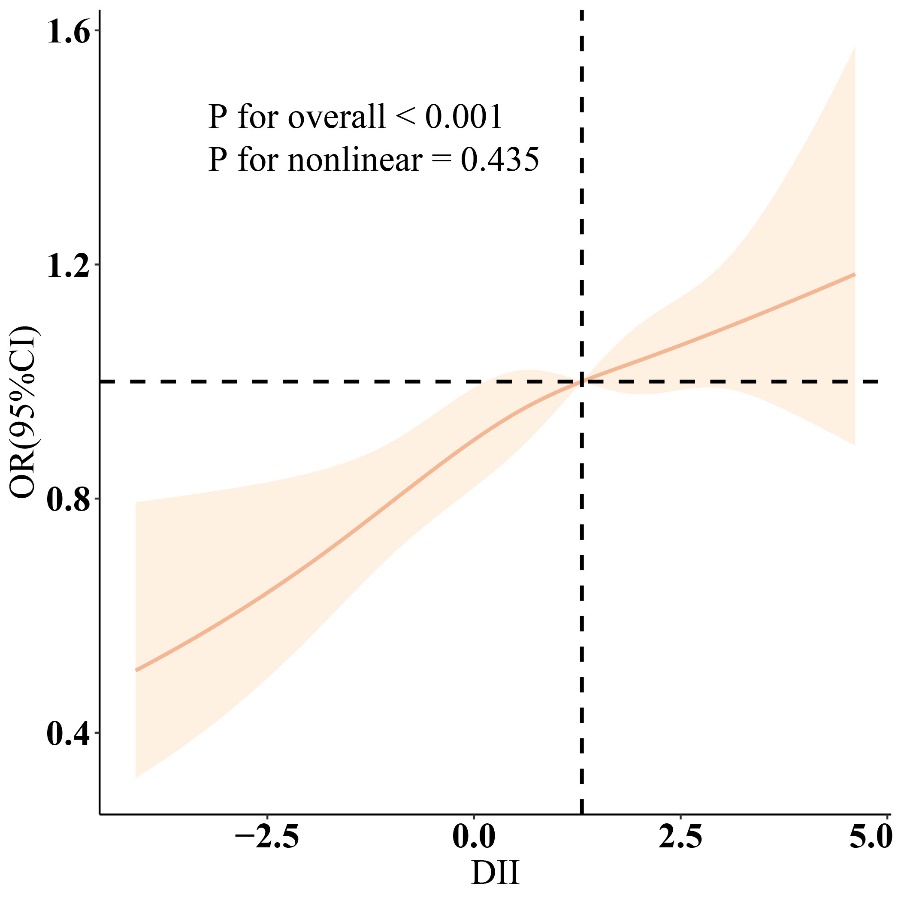


**Figure S2.** Restricted cubic spline curves for the association between the DII and COPD. Adjusted for Adjusted for age, gender, education level, marital, PIR, race, obesity, smoking, drinking, sleep disorder, CCI.

**Table S1.** Description of covariates

| Covariates | Description in NHANES |
| --- | --- |
| Age | Divided into three groups: 20-40 years old, 41-60  years old, >60 years old |
| Gender | Male and Female |
| Race | Mexican American, Non-Hispanic Black, Non-Hispanic White, Other Race |
| Educational level | Below high school, High School or above |
| Marital status | Yes: Married/Living with partner |
| PIR | Poor: <1.3; Not Poor:>=1.3 |
| Obesity | Yes: BMI>=30 |
| Smoking | Smoking status was grouped into never smoker (defined as <100 cigarettes in a lifetime), current smoker (defined as ≥100 cigarettes in a lifetime), and former smoker (defined as ≥100 cigarettes and had quit smoking) |
| Drinking | Drinking history was defined as consuming at least 12 drinks in any year |
| Sleep disorder | Participants were asked whether they had sleep disorder |
| CCI | <1, Participants who do not report whether they suffer from a certain disease as healthy |

PIR, Ratio of family income to poverty; CCI, Charlson Comorbidity Index.

**Table S2.** Charlson Comorbidity Index Scores of various diseases included in this study.

| **Disease** | **Score** |
| --- | --- |
| Diabetes | 1 |
| Diabetic retinopathy | 2 |
| Kidney failure | 2 |
| Kidney stones | 2 |
| Heart failure | 1 |
| Stroke | 1 |
| Hepatopathy | 2 |
| Rheumatoid arthritis | 1 |
| Bladder cancer | 2 |
| Bone cancer | 2 |
| Brain cancer | 2 |
| Breast cancer | 2 |
| Cervical cancer | 2 |
| Colon cancer | 2 |
| Esophageal cancer | 2 |
| Gallbladder carcinoma | 2 |
| Kidney cancer | 2 |
| Tracheal carcinoma | 2 |
| Leukemia | 2 |
| Liver cancer | 2 |
| Lung cancer | 2 |
| Lymphomas | 2 |
| Melanoma | 2 |
| Oral cancer | 2 |
| Never cancer | 2 |
| Ovarian cancer | 2 |
| Pancreatic cancer | 2 |
| Prostatic cancer | 2 |
| Rectal cancer | 2 |
| Skin cancer(non-melanoma) | 2 |
| Other skin cancer | 2 |
| Soft tissue cancer | 2 |
| Stomach cancer | 2 |
| Testicular cancer | 2 |
| Thyroid cancer | 2 |
| Endometrial cancer | 2 |
| Other cancer | 2 |

**Table S3.** Multivariate linear regression of NHHR and DII.

|  | β | 95%CI | P-value |
| --- | --- | --- | --- |
| NHHR - DII | 0.10 | (0.07,0.12) | <0.001 |

Adjusted for Adjusted for age, gender, education level, marital, PIR, race, obesity, smoking, drinking, sleep disorder, CCI.

**Table S4.** Association between NHHR, DII and COPD using the multiple imputation data results.

| **Characteristics** | **Model 1 [OR (95% CI)]** | **P** | **Model 2 [OR (95% CI)]** | **P** | **Model 3 [OR (95% CI)]** | **P** |
| --- | --- | --- | --- | --- | --- | --- |
| **NHHR** |  |  |  |  |  |  |
| **Continues** | 1.08 (1.06, 1.11) | <0.001 | 1.11 (1.08, 1.14) | <0.001 | 1.09 (1.06, 1.12) | <0.001 |
| Q1 | 1 (ref.) |  | 1 (ref.) |  | 1 (ref.) |  |
| Q2 | 1.22 (1.11, 1.33) | <0.001 | 1.24 (1.13, 1.36) | <0.001 | 1.23 (1.12, 1.35) | <0.001 |
| Q3 | 1.25 (1.14, 1.37) | <0.001 | 1.31 (1.20, 1.44) | <0.001 | 1.30 (1.18, 1.42) | <0.001 |
| Q4 | 1.42 (1.27, 1.58) | <0.001 | 1.53 (1.38, 1.70) | <0.001 | 1.46 (1.31, 1.62) | <0.001 |
| ***P for trend*** | <0.001 |  | <0.001 |  | <0.001 |  |
| **DII** |  |  |  |  |  |  |
| **Continues** | 1.10 (1.07, 1.12) | <0.001 | 1.08 (1.06, 1.11) | <0.001 | 1.06 (1.04, 1.09) | <0.001 |
| Q1 | 1 (ref.) |  | 1 (ref.) |  | 1 (ref.) |  |
| Q2 | 1.18 (1.10, 1.27) | <0.001 | 1.18 (1.09, 1.27) | <0.001 | 1.15 (1.06, 1.24) | <0.001 |
| Q3 | 1.37 (1.25, 1.50) | <0.001 | 1.33 (1.22, 1.46) | <0.001 | 1.26 (1.15, 1.37) | <0.001 |
| Q4 | 1.55 (1.34, 1.79) | <0.001 | 1.46 (1.27, 1.67) | <0.001 | 1.32 (1.16, 1.50) | <0.001 |
| ***P for trend*** | <0.001 |  | <0.001 |  | <0.001 |  |

Model 1: no covariates were adjusted;

Model 2: age, gender, education level, marital, PIR, and race were adjusted;

Model 3: age, gender, education level, marital, PIR, race, obesity, smoking, drinking, sleep disorder, CCI were adjusted;

Abbreviation: NHHR, Non-high-density lipoprotein cholesterol (non-HDL-C) to high-density lipoprotein cholesterol (HDL-C) ratio; COPD, Chronic obstructive pulmonary disease; DII, dietary inflammatory index; CCI, Charlson Comorbidity Index; PIR, Ratio of family income to poverty; OR, odds ratio; CI, confidence interval.

**Table S5.** Multivariate linear regression of NHHR and DII using the multiple imputation data results.

|  | β | 95%CI | P-value |
| --- | --- | --- | --- |
| NHHR - DII | 0.07 | (0.05,0.09) | <0.001 |

Adjusted for Adjusted for age, gender, education level, marital, PIR, race, obesity, smoking, drinking, sleep disorder, CCI.

**Table S6.** Association between NHHR, DII and COPD excluding participants with asthma.

| **Characteristics** | **Model 1 [OR (95% CI)]** | **P** | **Model 2 [OR (95% CI)]** | **P** | **Model 3 [OR (95% CI)]** | **P** |
| --- | --- | --- | --- | --- | --- | --- |
| **NHHR** |  |  |  |  |  |  |
| **Continues** | 1.11 (1.05, 1.18) | <0.001 | 1.18 (1.11, 1.25) | <0.001 | 1.09 (1.02, 1.17) | 0.013 |
| Q1 | 1 (ref.) |  | 1 (ref.) |  | 1 (ref.) |  |
| Q2 | 1.07 (0.83, 1.37) | 0.600 | 1.09 (0.83, 1.43) | 0.500 | 1.00 (0.75, 1.34) | 0.900 |
| Q3 | 1.23 (0.95, 1.59) | 0.110 | 1.29 (0.98, 1.72) | 0.073 | 1.15 (0.86, 1.54) | 0.400 |
| Q4 | 1.53 (1.20, 1.95) | <0.001 | 1.80 (1.39, 1.33) | <0.001 | 1.36 (1.02, 1.81) | 0.037 |
| ***P for trend*** | <0.001 |  | <0.001 |  | 0.022 |  |
| **DII** |  |  |  |  |  |  |
| **Continues** | 1.20 (1.14, 1.27) | <0.001 | 1.15 (1.09, 1.22) | <0.001 | 1.10 (1.04, 1.16) | 0.001 |
| Q1 | 1 (ref.) |  | 1 (ref.) |  | 1 (ref.) |  |
| Q2 | 1.54 (1.15, 2.08) | 0.004 | 1.53 (1.11, 2.12) | 0.010 | 1.46 (1.03, 2.06) | 0.033 |
| Q3 | 1.70 (1.31, 2.21) | <0.001 | 1.53 (1.15, 2.04) | 0.004 | 1.39 (1.02, 1.87) | 0.034 |
| Q4 | 2.40 (1.70, 3.21) | <0.001 | 2.06 (1.52, 2.79) | <0.001 | 1.67 (1.24, 2.26) | 0.001 |
| ***P for trend*** | <0.001 |  | <0.001 |  | 0.001 |  |

Model 1: no covariates were adjusted;

Model 2: age, gender, education level, marital, PIR, and race were adjusted;

Model 3: age, gender, education level, marital, PIR, race, obesity, smoking, drinking, sleep disorder, CCI were adjusted;

Abbreviation: NHHR, Non-high-density lipoprotein cholesterol (non-HDL-C) to high-density lipoprotein cholesterol (HDL-C) ratio; COPD, Chronic obstructive pulmonary disease; DII, dietary inflammatory index; CCI, Charlson Comorbidity Index; PIR, Ratio of family income to poverty; OR, odds ratio; CI, confidence interval.

**Table S7.** Multivariate linear regression of NHHR and DII excluding participants with asthma.

|  | β | 95%CI | P-value |
| --- | --- | --- | --- |
| NHHR - DII | 0.10 | (0.07,0.13) | <0.001 |

Adjusted for Adjusted for age, gender, education level, marital, PIR, race, obesity, smoking, drinking, sleep disorder, CCI.

**Table S8.** Association between NHHR, DII and COPD further adjusted for Triglycerides, LDL-C, HDL-C, LDL-C, and Glycohemoglobin.

| **Characteristics** | **OR (95% CI)]*** | **P** |
| --- | --- | --- |
| **NHHR** |  |  |
| **Continues** | 1.09 (1.01, 1.19) | 0.031 |
| Q1 | 1 (ref.) |  |
| Q2 | 1.11 (0.78, 1.45) | 0.200 |
| Q3 | 1.17 (0.81, 1.45) | 0.220 |
| Q4 | 1.55 (1.20, 1.92) | <0.001 |
| ***P for trend*** | <0.001 |  |
| **DII** |  |  |
| **Continues** | 1.06 (1.00, 1.13) | 0.047 |
| Q1 | 1 (ref.) |  |
| Q2 | 1.27 (0.93, 1.74) | 0.130 |
| Q3 | 1.14 (0.85, 1.54) | 0.400 |
| Q4 | 1.42 (1.04, 1.94) | 0.028 |
| ***P for trend*** | 0.030 |  |

*****Age, gender, education level, marital, PIR, race, obesity, smoking, drinking, sleep disorder, CCI, Triglycerides, LDL-C, HDL-C, LDL-C, and Glycohemoglobin were adjusted.

Abbreviation: NHHR, Non-high-density lipoprotein cholesterol (non-HDL-C) to high-density lipoprotein cholesterol (HDL-C) ratio; COPD, Chronic obstructive pulmonary disease; DII, dietary inflammatory index; CCI, Charlson Comorbidity Index; PIR, Ratio of family income to poverty; OR, odds ratio; CI, confidence interval.
